# PD-1/PD-L1 Expression Could Be a Prognostic Factor in Patients with Acute Myeloid Leukemia

**DOI:** 10.5152/eurasianjmed.2026.251217

**Published:** 2026-07-07

**Authors:** Gizem Varkal, Emel Gürkan, Emine Kılıç Bağır, Burak Mete

**Affiliations:** 1 Department of Internal Medicine, Çukurova University Faculty of Medicine, Adana, Türkiye; 2 Division of Hematology, Department of Internal Medicine, Çukurova University Faculty of Medicine, Adana, Türkiye; 3 Department of Pathology, Çukurova University Faculty of Medicine, Adana, Türkiye; 4 Division of Public Health, Department of Internal Medicine, Çukurova University Faculty of Medicine, Adana, Türkiye

**Keywords:** Acute myeloid leukemia, immune checkpoints, prognosis

## Abstract

**Background:**

In this research, PD-1 and PD-L1 expression patterns were examined to determine their prevalence and prognostic relevance among patients with acute myeloid leukemia (AML).

**Methods:**

Ninety-nine patients diagnosed with AML at the Hematology Department of Çukurova University Medical School between March 2009 and June 2017 were retrospectively analyzed. The expression levels of PD-1 and PD-L1 in bone marrow biopsy samples were determined using immunohistochemical methods. Their correlations with clinical and laboratory characteristics, treatment response, and survival data were analyzed.

**Results:**

PD-1/PD-L1 positivity (≥ 5%) was observed in 9.1% of the cohort (n = 9). At the time of diagnosis, patients showing PD-1/PD-L1 expression exhibited significantly lower hemoglobin levels than those without expression (7.25 g/dL vs 8.80 g/dL; *P* = .017). Elevated PD-1/PD-L1 expression correlated with poorer overall survival, with a median OS of 11 months (95% CI: 5.9-16.1) compared to 24 months (95% CI: 13.4-34.6) in the non-expressing group (*P* = .033).

**Conclusion:**

Although PD-1/PD-L1 expression was relatively infrequent among AML patients, positive cases had significantly poorer survival. These findings suggest that PD-1/PD-L1 expression may be associated with poorer clinical outcomes in AML; however, larger studies are needed to clarify its prognostic relevance.

**Main Points**

- PD-1/PD-L1 upregulation was detected in 9.1% of patients with acute myeloid leukemia, indicating that immune checkpoint activation occurs in a small but clinically relevant subset.
- Individuals expressing PD-1/PD-L1 had reduced hemoglobin values at the time of diagnosis, which may indicate a link with increased disease severity.
- An inverse association was identified between PD-1/PD-L1 levels and overall survival duration in this study group; nonetheless, the restricted sample size of positive cases necessitates a conservative interpretation of the data.

## Introduction

Checkpoint blockade immunotherapy is an innovative cancer therapeutic approach that has demonstrated efficacy across various cancer types, including in patients with advanced disease.^1^ The therapeutic application of immune checkpoint proteins (ICPs) in cancer management has led to remarkable clinical outcomes in patients with Hodgkin lymphoma (HL).^2^ Based on the data indicating the efficacy of ICPs in solid tumors and HL, there has been a significant interest in the potential role of ICPs in other hematological malignancies including acute leukemias. Despite marked improvements in acute myeloid leukemia (AML) treatment, the outcomes are still poor, especially in individuals with severe risk factors. As in other cancers, prognosis in AML also depends on several factors including immune response mechanisms and tumor microenvironment (TM). One of the pathways contributing to tumor immune escape involves the upregulation of co-inhibitory receptors and their binding partners.^3^ Antitumor immunity is systematically suppressed through the interaction between PD-1, expressed on activated monocytes and T/B lymphocytes, and its ligand PD-L1, which resides on antigen-presenting or malignant cells; this mechanism facilitates immune evasion by neoplastic cells. Consequently, previous literature has established that aberrant PD-1 upregulation on these cells severely impairs T-cell-mediated reactivity against tumors.^4^ Targeting the inhibitory immune checkpoint mechanisms utilized by cancer cells to escape immune surveillance has emerged as an effective strategy for restoring antitumor immunity over the past decade. However, studies involving immune dysfunction in acute leukemias are scarce. The present study focuses on the quantification of PD-1 and PD-L1 inside the AML tumor microenvironment, aiming to establish their potential associations with the clinical behavior of the disease and patient survival.

## Material and Methods

Ninety-nine patients with AML who were diagnosed in the Hematology Department of Çukurova University Medical School between March 2009 and June 2017 were included. The Local Ethics Committee of Çukurova University accepted the study protocol. (Approval date: July 7, 2017, no: 66/4). Ethical compliance was maintained throughout the study in accordance with the Declaration of Helsinki, and signed consent forms were collected from all individuals. The demographic data and laboratory values of the patients were documented. Acute myeloid leukemia diagnosis was confirmed when 20% or more blasts were identified in the peripheral blood or bone marrow, accompanied by relevant cytogenetic alterations. The myeloid origin of leukemic cells was confirmed using routine diagnostic procedures, including flow cytometry and immunohistochemical staining performed at the time of diagnosis. Initial treatment for the entire cohort consisted of the conventional "7+3" regimen. However, patients with acute promyelocytic leukemia (APL) were managed with induction therapy that combined all-trans retinoic acid (ATRA) and anthracycline-based protocols. For non-APL acute myeloid leukemia (AML) cases requiring salvage therapy, the FLAG± Idarubicin regimen was administered. Criteria for achieving complete remission (CR) required bone marrow blasts to fall below 5% without evidence of Auer rods, extramedullary disease, or circulating blasts. Additionally, hematologic recovery guidelines necessitated a platelet count of 100× 10^9^/L, an absolute neutrophil count (ANC) of at least 1x10^9^/L. Partial hematologic recovery (CRp) referred to cases fulfilling all CR criteria except for partial recovery of blood counts, with ANC ≥ 0.5 × 10^9^/L and platelet count ≥ 50 × 10^9^/L. When remission criteria were met but neutropenia (ANC <1 × 10^9^/L) or thrombocytopenia (platelet count <100 × 10^9^/L) persisted, the response was categorized as incomplete hematologic recovery (CRi). Patients who failed to achieve CR, CRp, or CRi following 2 cycles of intensive induction therapy were considered refractory.

Immunohistochemical analysis was performed on archived BM biopsy specimens to determine the expression profiles of both PD-1 and PD-L1. Immunohistochemical analysis was conducted on 5 µm slices of formalin-fixed, paraffin-embedded BM biopsy specimens.

**Figure 1.**
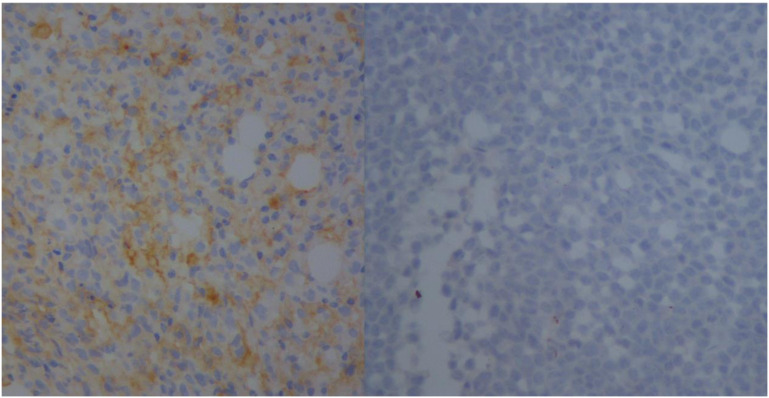
Immunohistochemical staining patterns of PD-L1 expression in bone marrow biopsy samples. Left: PD-L1-positive staining. Right: PD-L1-negative staining. Original magnification ×200.

The detection of PD-1 and PD-L1 was carried out using specific monoclonal antibodies, EPR4877 (Abcam, USA) and E1L3N (Cell Signaling, USA), respectively. Staining procedures were performed on the automated Ventana BenchMark XT system. A sample was considered positive when ≥ 5% of tumor cells showed cytoplasmic and/or membranous staining. This cut-off was selected according to previous immunohistochemical studies evaluating PD-1/PD-L1 expression in hematologic malignancies.^5^ Positivity for leukemic cells and the microenvironment was defined as staining over 5% of the population. Staining intensity was not separately scored, and the evaluation was based primarily on the proportion of positively stained leukemic cells within the bone marrow biopsy specimens (Figure 1).Both H&E and immunohistochemical samples were evaluated by 2 separate pathologists. Ethical approval for this study was formally granted by the local institutional review board.

### Statistical Analysis

The IBM SPSS Statistics software (version 21; IBM Corp., Chicago, IL, USA) provided the platform for all mathematical computations. Non-parametric instruments were selected due to the non-normal skewness of the parameters. Specifically, inter-group variances were scrutinized using the Mann–Whitney *U* test (for 2 groups) and the Kruskal–Wallis test (for multiple groups). Spearman’s rank test quantified correlations among continuous variables, and the chi-square test evaluated categorical data dependencies. Descriptive presentations included mean ± SD or median (interquartile range [IQR]) based on normality, alongside frequencies (%) for categorical indicators. Finally, Kaplan–Meier estimates tracked survival starting from the primary diagnosis date, with log-rank testing confirming variance between strata. A 2-tailed defined statistical significance.

## Results

The study cohort comprised 99 participants with a median age of 51 years (IQR: 28; range: 17-87).

In terms of sex distribution, the group included 50 male (50.5%) and 49 female (49.5%) subjects.

De novo AML was diagnosed in 88 (89%) of the patients, whereas 11 (11%) of the patients had secondary AML either with a prior MDS (6) or MPN (5). Among patients diagnosed with de novo AML, 19 cases (19.2%) were identified as having APL.

Table 1 summarizes the demographic features of the study population.

Seventeen of the patients died during induction therapy. One of the patients died from septic shock before induction therapy. Nineteen patients underwent allogeneic stem cell transplantation (SCT).

At a median follow-up duration of 14 months (range: 1-118 months), 37 patients (37.3%) had died. The 24-month overall survival rates for AML patients in the good-, intermediate-, and poor-risk groups were 84.0%, 79.7%, and 49.2%, respectively (*P* = .053). Thus, patients within the poor-risk group had a survival disadvantage compared to good and intermediate-risk patients as expected.

PD-1 and PD-L1 expression were also evaluated separately. PD-1 positivity was detected in 4 patients, whereas PD-L1 positivity was observed in 9 patients. All PD-1-positive cases demonstrated concurrent PD-L1 positivity. Due to the limited number of positive cases in each subgroup, subsequent analyses were performed using combined PD-1/PD-L1 expression status. Therefore, survival and clinicopathological analyses were conducted using the combined PD-1/PD-L1 positivity status in order to avoid statistically unreliable subgroup comparisons.

Overall, PD-1/PD-L1 positivity (≥ 5% staining) was detected in 9 of the 99 AML patients (9.1%) included in the study. Within the PD-1 ± PD-L1 expressing cohort, 2 individuals had APL. Because the number of APL patients with PD-1/PD-L1 positivity was very limited, a separate subgroup analysis for APL could not be performed. According to risk classification, 2 of the patients with positive expression were in good risk, 4 intermediate and 1 was in poor risk groups.

**Figure 2.**
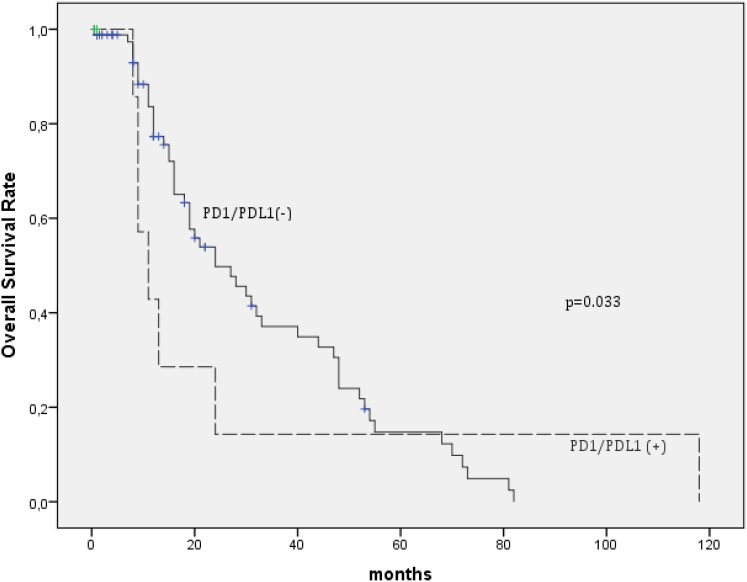
Overall survival of patients with AML according to PD-1/PD-L1 expression.

When the clinical data of expressors and non-expressors were compared, patients with positive PD-1/PD-L1 expression were younger (*P* = .027) and had significantly lower hemoglobin levels at diagnosis (*P* = .017) (Table 2). Induction therapy response rates were comparable between PD-1/PD-L1 positive and negative AML patients, showing no significant statistical divergence. Elevated PD-1 ± PD-L1 levels were linked to an unfavorable prognosis for overall survival, as the median OS was 11 months (95% CI: 5.9-16.1) among expressors, compared to 24 months (95% CI: 13.4-34.6) among non-expressors (*P* = .033) (Figure 2).

## Discussion

AML represents a heterogeneous malignancy in which immature hematopoietic progenitors, blocked at an early differentiation stage, accumulate within the BM and infiltrate peripheral tissues. Conventional chemotherapy with the standard “7+3 regimen” of cytarabine plus anthracycline has been the mainstay of the treatment for over 50 years. Despite its therapeutic potential, sustained clinical remission is achieved in only a limited subset of patients receiving this regimen. Most of the patients, especially those with high risk, experience frequent relapses or refractoriness with a dismal disease course. Therefore, improving treatment success, reducing morbidity, and extending survival have become important goals for this group of diseases. Following standard treatments, SCTs have been performed in eligible patients. The effective execution of allogeneic SCT, leveraging its graft versus leukemia effect to eliminate leukemia cells in AML, has heightened interest in the application of immune treatments for this patient population. The effects of immune therapies have aroused curiosity in the medical world, and research has been conducted on many malignant patient groups. During the past decade, blockade of the PD-1 signaling axis has demonstrated remarkable efficacy across multiple solid malignancies and in Hodgkin lymphoma (HL).^6^^,^^7^

Leukemic cells, derived from an immunological lineage, typically exhibit immune checkpoint molecules that serve as targets for immune checkpoint inhibition.^8^ These inhibitory receptors are known as cytotoxic T-lymphocyte-associated protein 4 (CTLA4) and PD-1.^8^ Experimental mouse studies on hematologic and solid tumors revealed PD-1 expression and a decline in tumor size after PD-1/PD-L1 inhibition.^9^ The results of these experimental studies justified conducting clinical studies. CTLA-4 and PD-1, which act as ICPs of T-cell immune activity, have been evaluated in clinical trials of hematologic cancers, including AML.^10^ The aim was to examine the PD1-PDL1 pathway and its prognostic significance in the AML patient group based on both experimental and clinical studies. The findings show that 9% of the patients with AML had a significant level of PD-1/PD-L1 expression. In the cohort, PD-1/PD-L1 expression appeared to be associated with poorer overall survival.

The clinical trial by Chen et al. is particularly notable in the literature. In a study by Chen et al.^11^, RNA-seq and mutation profiles from 176 AML cases in the TCGA database were analyzed and validated using bone marrow samples from 62 de novo AML patients. According to the authors, upregulated levels of PD-1 along with its ligands, PD-L1 and PD-L2, independently correlated with a decline in overall survival among AML patients.^11^ Other research has shown that elevated PD-L1 levels may be linked to more severe disease behavior and worse clinical outcomes in AML.^12^^-16^ Fevery et al. have demonstrated previously that CTLA-4 blockade induces a host-derived anti-leukemic effect.^17^ Numerous studies have highlighted the key involvement of PD-1 in tumor immune escape and T-cell exhaustion. Blocking the CTLA-4 and PD-1/PD-L1 axes has been shown to improve anti-leukemic responses, resulting in tumor reduction and prolonged survival in experimental mouse models.^18^^-^^21^ In a clinical investigation conducted by Ruan et al. (2023), the prognostic relevance of PD-1 expression was assessed. PD-L1 levels were determined in peripheral blood by flow cytometry among 57 AML and 45 ALL patients, along with 28 healthy controls. According to the findings, upregulated PD-L1 expression served as an adverse prognostic indicator, correlating with shortened patient survival.^22^ In the study, survival was found to be lower in patients with PD1/PDL1 expression.

Previous studies have suggested that high PD-1 and CTLA-4 expression may be associated with a more aggressive disease course in leukemia.^23^ Although it is unlikely to derive such conclusions from the study, patients with PD-1/PD-L1 expression in the cohort were younger and presented with more depressed hemoglobin levels. However, patients with PD-1/PD-L1 expression showed shorter overall survival in the cohort. Given the limited number of PD-1/PD-L1 positive cases, these findings should be interpreted cautiously. Nevertheless, PD-1/PD-L1 upregulation appeared to be associated with a less favorable clinical course in AML patients.

### Limitations

Despite its contributions, this study has several limitations that should be considered. First, the retrospective design and the use of archived biopsy samples limited the ability to perform a more comprehensive evaluation of the immune microenvironment. In addition, the relatively small cohort size and the low number of PD-1/PD-L1-positive cases may have reduced the statistical power of the analyses. The inclusion of APL patients, a biologically and clinically distinct AML subtype, may also have contributed to cohort heterogeneity. Another limitation was the lack of detailed analysis of immune cell populations within the bone marrow microenvironment, including CD3⁺, CD4⁺, and CD8⁺ T-cell subsets. Therefore, the mechanisms underlying immune escape could not be fully evaluated. Larger prospective studies with more detailed immune profiling are needed to better clarify the prognostic relevance of the PD-1/PD-L1 pathway in AML.

## Conclusion

PD-1/PD-L1 upregulation on leukemic cells may be associated with adverse clinical outcomes in AML. To firmly establish these prognostic associations, verification through expansive, forward-looking cohort investigations is still necessary. Further studies in order to better understand TM and its correlations, and also clinical trials involving antibodies targeting PD-1 pathways, i.e., immune checkpoint inhibitors in AML patients, are needed. In the future, understanding the PD-1/PD-L1 pathway with larger studies will provide hope for more successful treatment.

## Figures and Tables

**Table 1.** Demographic Characteristics of the Patients

n (%)	
Gender (male)	50 (50.5)
Age(years)^a^	51 (28*)
AML subtype (n/%)	
De novo	88 (89)
Secondary	11 (11)
AML risk group*	
Good	19 (19.2)
Intermediate	40 (40.4)
Poor	16 (16.2)
Not classified/unavailable	24 (24.2)
Leukocyte count (10^9^/L)^a^	7.29 (30.94*)
Hb (g/dL)^a^	8.70 (3*)
Plt(10^9^/µL)^a^	41 (78*)
LDH (IU/L)^a^	309 (364*)
Extramedullary involvement	12 (13.3)
Blast percentage by flow cytometry^a^	41.85 (43.93*)
Response to induction therapy n (%)	
Yes	43 (43.4)
No	38 (38.3)
Early death	18 (18.18)
Follow-up ((mean ± SD) months)^b^	14 ± 22
Mean survival ((mean ± SD) months)	24 ± 3.6

AML, acute myeloid leukemia; IQR, interquartile range; LDH, lactate dehydrogenase.

*aData are presented as median IQR.: ^b^Data are presented as mean ± SD.

Interquartile range (IQR) AML risk according to ELN 2017 classification LDH normal range 115-248 IU/L.

**Table 2.** Comparison of Clinical Characteristics of Patients According to PD-1 ± PD-L1 Expression

	PD-1/PD-L1 (+) (n = 9)	PD-1/PD-L1(−) (n = 90)	*P*
Age(years)	32 (33*)	53 (26*)	0.027
Female/male	4/5	45/45	NS
AML subtype			
De novo (non-APL)	7	62	NS
Secondary	0	11	
De novo APL	2	17	
AML risk group			
Good	2	17	NS
Intermediate	4	36	
Poor	1	15	
Not classified/unavailable	2	22	
Response to induction therapy			
Yes	3	40	NS
No	3	35	
Hemoglobin (g/dL)^a^	7.25 (4*)	8.80 (3*)	0.017
Leukocyte count (10^9^ /L)	1.19 (19.59*)	6.23 (30.13*)	NS
Blast percentage by flow cytometry^a^	56.26 (31.57*)	39.78 (44.49*)	NS
LDH (IU/L)^a^	247 (460*)	317 (360*)	NS
Survival			
Alive	5	67	0.033
Death	4	33	

IQR, interquartile range; AML, acute myeloid leukemia; LDH, lactate dehydrogenase.

^a^Data are given as median (IQR).

## Data Availability

The data that support the findings of this study are available on request from the corresponding author.
